# Systematic review of feedback literacy instruments for health professions students

**DOI:** 10.1016/j.heliyon.2024.e31070

**Published:** 2024-05-10

**Authors:** Mohamad Nabil Mohd Noor, Sahar Fatima, Jessica Grace Cockburn, Muhammad Hibatullah Romli, Vinod Pallath, Wei-Han Hong, Jamuna Vadivelu, Chan Chong Foong

**Affiliations:** a Medical Education and Research Development Unit, Universiti Malaya, Kuala Lumpur, Malaysia; b Department of Surgical Oncology, University Health Network, Toronto, Canada; c Department of Rehabilitation Medicine, Universiti Putra Malaysia, Serdang, Malaysia; d Jeffrey Cheah School of Medicine and Health Sciences, Monash University Malaysia, Bandar Sunway, Selangor, Malaysia

## Abstract

Successfully managing and utilizing feedback is a critical skill for self-improvement. Properly identifying feedback literacy level is crucial to facilitate teachers and learners especially in clinical learning to plan for better learning experience. The present review aimed to gather and examine the existing definitions and metrics used to assess feedback literacy (or parts of its concepts) for health professions education. A systematic search was conducted on six databases, together with a manual search in January 2023. Quality of the included studies were appraised using the COSMIN Checklist. Information on the psychometric properties and clinical utility of the accepted instruments were extracted. A total 2226 records of studies were identified, and 11 articles included in the final analysis extracting 13 instruments. These instruments can be administered easily, and most are readily accessible. However, ‘appreciating feedback’ was overrepresented compared to the other three features of feedback literacy and none of the instruments had sufficient quality across all COSMIN validity rating sections. Further research studies should focus on developing and refining feedback literacy instruments that can be adapted to many contexts within health professions education. Future research should apply a rigorous methodology to produce a valid and reliable student feedback literacy instrument.

## 1 Introduction

### 1.1 Feedback in health professions education

The feedback process is crucial for aiding learners in realizing their present capabilities, offering a chance to enhance their competencies [1]. Uniquely in health profession education, students need to apply feedback to improve knowledge, skills and attitudes in experiential learning settings, for instance the clinics [2]. Learners are expected to receive feedback opportunistically, in settings (e.g. emergency rooms, operating theatres) which may not be as conducive for students to appreciate and analyze the feedback [[3], [4], [5]]. Plus, health profession students are subjected to clinical rotations in different hospital departments, exposing them to short-term, changing supervisors who may have different feedback formats depending on their speciality [6,7]. Ultimately, these learners need to evaluate and internalize feedback to improve their competency for the sake of patient safety [8,9]. Feedback literacy can help address the unique challenges posed by health professions education practices of feedback and enhance the efficacy of the process.

### 1.2 Definition and features of feedback literacy

Feedback literacy is the ability to comprehend, analyze, and apply feedback in order to strengthen one's competencies [10]. Feedback-literate learners are characterized by the capacities and dispositions to appreciate the feedback process, make judgments on feedback, regulate their emotions in the face of criticisms and taking the initiative to apply these feedback [10,11]. Therefore, fostering feedback literacy among learners empowers them to take responsibility and collaborate with feedback providers to determine the necessary steps in enhancing their competencies [[11], [12], [13]]. Thus, learners may be better equipped to manoeuvre the circumstances of receiving feedback in the context of health professions education.

### 1.3 Measurement of feedback literacy

Considering that feedback literacy contributes to learning, its measurement is needed as an objective way for educators to evaluate the students' capacities to participate in the feedback process [14,15]. Several tools have been developed to measure feedback literacy for health professions students [16,17]. These instruments were developed based on Carless and Boud's conceptualization of feedback literacy [10]. However, these instruments have not been formally critiqued for its validity and alignment with the concept of feedback literacy [15]. Also, there are instruments that do not assess feedback literacy as the exact concept, but they evaluate related constructs that encompass its features [18,19]. For example, the ‘Feedback Survey Instrument’ [20] measures medical students' attitudes and experiences receiving feedback in a clinical rotation. Although these constructs do not measure feedback literacy in full, it still indicates students' understandings of the feedback process. By including instruments like this in a review, we can construct a deeper and comprehensive understanding of feedback literacy measurement in health professions education.

### 1.4 Aim and significance of this review

Since the development of a feedback literacy scale for medical residents in 2007 [21], it is a prime time for different instruments measuring feedback literacy to be reviewed and examined as the discussion on feedback literacy grows rapidly over recent years [11,[22], [23], [24]]. To the knowledge of the authors, no systematic review was conducted for the above purpose. By systematically reviewing existing literature, it is possible to assess in detail the concept, utility, and methodological qualities of feedback literacy instruments that have been used for health profession students. This review aims to gather and examine the existing definitions and metrics used to assess feedback literacy (or parts of its concepts) for health professions education. In turn, educators can make evidence-informed decisions when choosing feedback literacy scales that are most appropriate for their needs and students can self-evaluate to improve their competency in receiving and using feedback. Additionally, the findings from this review may serve researchers to further discuss feedback literacy measurement, specifically to the context of health professions education. For instance, whether the existing feedback literacy scales may be adopted, adapted, re-developed or re-validated for the use among health professions students.

## 2 Materials and methods

This review was guided by the recommendations of Cook and West [25] for its practical step-by-step guide, specific and sensitive to the practices in health professions context. The guide has been widely used in systematic reviews in the health professions context [[26], [27], [28], [29], [30], [31], [32], [33], [34], [35], [36]]. This review was registered on INPLASY (registration number: INPLASY202370008) and had been reported according to the PRISMA guidelines [37], which has been referred to as the standard for systematic review reporting.

### 2.1 Study identification

A review was systematically conducted on the following six databases: Scopus, Medline, Web of Science, Education Research Complete, Cumulative Index to Nursing and Allied Health Literature (CINAHL) Complete, and Psychology and Behavioural Sciences Collection. The main keywords used were health, feedback literacy, psychometric properties, and instrument. The keywords for the electronic search were selected using the PICO format and were expanded by including synonyms and relevant terms based on published reviews and Medical Subject Heading (MeSH) terms to ensure a comprehensive search. Also, a pilot search was carried out to identify keywords that can collect research papers exploring feedback literacy or any of its features, regardless of the year of publications. Commands such as Boolean operators and truncations were applied when necessary. The full list of the search string can be referred to in the supplementary material. In addition to searching the electronic databases, a manual search was performed. References and citations of relevant articles were screened for more potential articles [25,38]. The study identification process was initiated on January 26, 2023.

### 2.2 Eligibility criteria

The studies identified in the search were assessed for eligibility based on the following criteria. Studies with the following criteria were included:

1. included health professions education of any level (undergraduate and/or postgraduate)
2. instrument measuring student feedback literacy or any of its features (regardless of the feedback provider, e.g. peers, patients, teachers) including adaptations, revalidation in a different context and replication studies
3. focusing on students' role in the feedback process i.e., behaviours and/or attitudes of students towards the feedback process
4. reporting any psychometric properties listed in the Consensus-based Standards for the Selection of Health Measurement Instruments (COSMIN) risk of bias checklist [39]
5. original study published in peer-reviewed journals.
6. published in any year.

Studies were further excluded if:

1. the instrument does not emphasize feedback literacy or its specific features (such as combination with other unrelated constructs or only mentions feedback literacy briefly)
2. developed instruments that do not focus on the students' roles in the feedback process, for example, feedback providers opinions on the feedback process
3. non-English publications
4 non-primary research articles, such as reviews, opinion pieces, editorials, or perspectives
5. gray literature (e.g., theses, dissertations, unpublished reports, conference presentations)
6. no available full text

### 2.3 Study selection

The first and second authors (doctoral candidates in medical education, with a master's degree in medical education, bachelor's degree in medicine and surgery, and have attended a systematic review training workshop) conducted the search by using the prepared terms. Then, duplicates were removed and screening of each record according to the eligibility criteria were done independently. First, the titles and abstracts were screened based on the eligibility criteria. In the event when the authors determined that the titles and/or abstracts meet these criteria, or when they were unsure, full texts of those studies were screened. When there was a disagreement between the two authors, a meeting was convened with the other authors to determine whether the study should be included or excluded. A repeat search of electronic databases was performed on May 26, 2023 to ensure this review accounts for recent publications.

### 2.4 Data extraction

Relevant data were extracted into four categories: general description, utility, constructs, and measurement properties of the included instruments. First, the instruments were described based on its author, year of publication, country, name of instrument, study design, number and level of participants, and summary of findings. Then, the utility of the instruments was extracted by its language, population, administration method, duration of administration, recall period, number of factors, number of items, response options, scoring method and accessibility. Next, details of the instrument's constructs were extracted including the method of concept elicitation, a brief description of the construct, category of construct (either it was knowledge, behaviour and/or perception of the students), feedback providers considered in the measurement and features of feedback literacy as compared to the concept outlined by Carless and Boud [10]. Finally, the measurement properties of each instrument were extracted and assessed as guided by the COSMIN Checklist [39]. Quality control is performed by conducting the inter-rater reliability on the pre-consensus agreement between the independent screener using the kappa analysis [40,41].

### 2.5 Quality appraisal

COSMIN Checklist was used to appraise the risk of bias and methodological quality of studies included in this review. This tool was chosen for its comprehensive and rigorous assessment of psychometric properties in systematic reviews. The checklist was initially created to evaluate Patient-Reported Outcome Measures (PROMs) [42] but has been adapted for systematic reviews of PROMs [39]. Although the checklist was initially created primarily for the patient population, it has also been utilized in systematic reviews of healthcare students and professionals [40,43], higher education students in general [44], and the public [45].

The quality appraisal using the COSMIN Checklist was guided by the user manual that are available online [46,47]. The checklist has ten ‘boxes’ of measurement properties under three headings and respective subheadings: content validity, internal structure, and the rest. The methodological quality was evaluated as either ‘inadequate’, ‘doubtful’, ‘adequate’, ‘very good’, or ‘not applicable’. Based on the ‘good measurement properties’, the results of the studies will be rated as either ‘insufficient’, ‘indeterminate’, or ‘sufficient’. Then, the quality of evidence was evaluated as ‘very low’, ‘low’, ‘moderate’, or ‘high’ according to the GRADE system. The two authors judged the methodological quality, results and quality of evidence using the COSMIN checklist and any disagreements will be addressed by discussing with the other authors.

### 2.6 Data synthesis

To synthesize the findings, a narrative approach was applied across the multiple instruments with varying constructs, conducted according to the guidelines of Popay, Roberts [48]. This approach has previously been applied to systematic psychometric reviews [43,44]. The instruments included in this review were compared based on their utility, constructs, and psychometric properties. Items in the instrument were compiled, coded and categorized into features of feedback literacy [10]. Then, a summary of the data was created. To ensure the credibility on the COSMIN rating, inter-rater reliability analysis on the pre-consensus decision between the two raters were conducted using the kappa analysis [40,41].

## 3 Results

### 3.1 Included studies

**Fig. 1:**
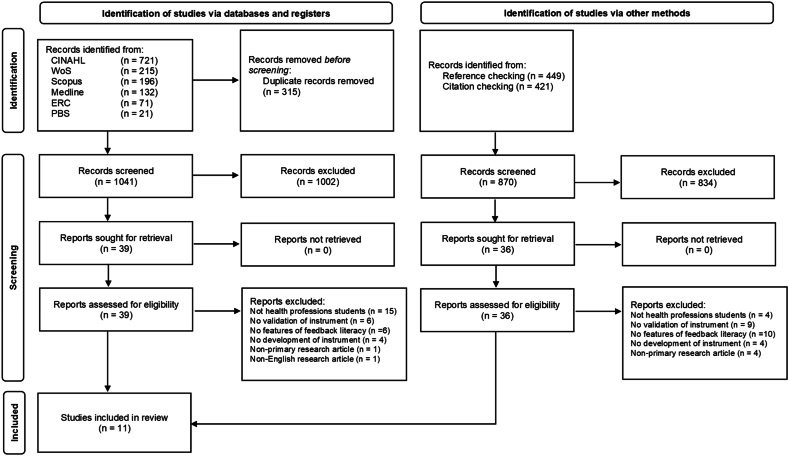
Flow diagram of study selection. *Abbreviations.* CINAHL, Cumulative Index to Nursing and Allied Health Literature; WoS, Web of Science; ERC, Education Research Complete; PBS, Psychology and Behavioral Sciences Collection.

The search process retrieved 2226 records of studies from both electronic databases and manual searches (Fig. 1). Of those, only 11 studies met the inclusion criteria and they were included in the review. This review was found to have adequate inter-rater reliability. Calculation of inter-rater reliability after full-text screenings and quality appraisal revealed a Cohen's kappa of 0.77, p < 0.001, and 0.72, p < 0.001 respectively, showing moderate reliability [41].

### 3.2 Health professions education student feedback literacy instruments

From the 11 included studies, 13 instruments were extracted (Table 1). These instruments were published in the years ranging from 2007 to 2021 and originated in different countries. Many of these instruments were newly developed, while the rest were adapted (translated into different languages and/or modification of items). In terms of study design, most of these instruments were published in quantitative studies, followed by mixed-methods studies and mixed data collection studies. Furthermore, nine out of the 13 instruments involved the medical discipline, two involved the dentistry discipline, with the rest being nursing, clinical psychology, and allied health. Additionally, the sample size involved in developing and validating these instruments ranged from 56 to 209 participants.

**Table 1:** Description of instruments.

Author	Year of Publication	Country	Instrument	Study Design	Participant	Finding
Al-Mously et al. [51],	2014	Saudi Arabia	Medical Students' Perceptions on the Quality of Feedback*	Quantitative	110 Year 5 and Year 6 medical students	Questionnaire was created based on review of past studies and piloted on a group of students.
Bing-You et al. [49],	2018	USA	FEEDME Provider	Mixed-Methods - Exploratory	132 medical students and residents	Two Delphi rounds modified and eliminated some items. Cognitive interviews with learners supported its comprehensibility. Exploratory factor analysis justified the construct validity with a two- and three-factor solution for the FEEDME Culture and FEEDME Provider instruments, respectively. Cronbach's alpha was higher than 0.80 for all factors.
Bing-You et al. [49],	2018	USA	FEEDME Culture	Mixed-Methods - Exploratory	139 medical students and residents	
Huancahuire-Vega et al. [50],	2021	Peru	FEEDME Culture - Spanish	Quantitative	139 Year 3, 4, 5 and 6 medical students	Modifications were made after focus group with medical students to improve comprehensibility. Exploratory factor analysis reported a two-factor structure. Cronbach's coefficient was higher than 0.80.
Goodrich et al. [55],	2021	USA	Corrective Feedback Acceptance and Synthesis in Supervision (CFASS)	Quantitative	73 clinical psychology master's students	Two doctoral students piloted the survey. Exploratory factor analysis supported a one-factor construct of the questionnaire. Cronbach's coefficient of the instrument was 0.86.
Janssen & Prins [21]	2007	Netherlands	Type of Information Sought	Quantitative	170 medical residents	Exploratory factor analysis supported a two-factor construct of the questionnaire. Cronbach's coefficient of the factors was more than 0.70. The hypothesis testing revealed a mixed findings based on the postulated hypothesis with different types of goal orientations.
Bose & Gijselaers [52]	2013	Switzerland	Type of Information Sought - German	Mixed-Methods - Explanatory	56 medical residents	Cronbach's alpha was 0.86 for the seeking of self-improvement information, and 0.66 for the seeking of self-validation information. Both type of information sought is significantly correlated with the monitoring method of feedback seeking.
Bose & Gijselaers [52]	2013	Switzerland	Frequency of Feedback Seeking - German	Mixed-Methods - Explanatory	56 medical residents	Cronbach's alpha was 0.53 for the inquiry method, and 0.72 for the monitoring method. Monitoring method is significantly correlated with both type of information sought.
Javed et al. [53],	2021	Pakistan	Dental Students' Perceptions on the Quality of Clinical Feedback*	Quantitative	64 final year dental students	The questionnaire was adapted from a previous study [65] and sent to senior dental researchers for comprehensiveness and comprehensibility
Milan et al. [20],	2011	USA	Feedback Survey Instrument	Mixed Data Collection	189 Year 3 medical students	Pilot study with 12 students was performed to ensure comprehensibility.
Nerali et al. [54],	2021	Malaysia	Dental Students' Perceptions towards Feedback during Clinical Training*	Mixed Data Collection	178 Year 3, 4 and 5 dental students	Questionnaire was created based on review of past studies and then validated by three content experts. Cronbach's alpha was 0.74.
Ossenberg et al. [16],	2020	Australia	Quality Feedback Inventory	Mixed-Methods - Exploratory	209 Year 3 nursing students	Items was generated based on a scoping review and then checked for comprehensiveness and comprehensibility with experts. Exploratory factor analysis revealed a three-factor structure and the Cronbach's alpha was 0.96.
Tripodi et al. [17],	2021	Australia	Feedback Literacy and Attitudes Survey	Quantitative	138 Year 1 Osteopathy students	The questionnaire were developed based on the feedback literacy framework by Carless and Boud [10] and the Cronbach's alpha was 0.86.

*Note:* Instrument titles marked with an asterisk (*) are given based on the article content as the instrument were not given a formal title in the original articles itself.

### 3.3 Utility of instruments

Twelve out of thirteen instruments are readily accessible from the research articles (appendix, methods, or results section), the utilities of which are listed in Table 2. The instruments comprise five to 37 items, with response options mainly being Likert scales. Ten instruments were developed in English, with the rest written in German and Spanish. Almost all the instruments require self-administration except for ‘Medical Students’ Perceptions on the Quality of Feedback’, which utilized structured interviews. Eleven instruments have involved students in their clinical rotations, and six have involved postgraduate students. Further, five instruments require participants to recall experiences from recent clinical rotations.

**Table 2:** Utility of instruments.

Instrument	Language	Population	Administration	Duration	Recall Period	Factor(s)	Items	Response Options	Scoring	Accessibility
Medical Students' Perceptions on the Quality of Feedback	English	Medical students in clinical rotations	Structured interview	15 min	Recently finished clinical rotation	5	37	4, 5-point Likert scale and dichotomous response	Individual scores of each items	May need to contact the author
FEEDME Provider	English	Medical students and residents	Self-administration	5–10 min	Daily encounters	3	13	5-point Likert scale	Total scores of each factors	Available as an appendix
FEEDME Culture	English	Medical students and residents	Self-administration	5–10 min	Current clinical rotation	2	16	5-point Likert scale	Total scores of each factors	Available as an appendix
FEEDME Culture - Spanish	Spanish	Medical students in clinical rotations	Self-administration	5–10 min	Current clinical rotation	2	11	5-point Likert scale	Total scores of each factors	Available as an appendix
Corrective Feedback Acceptance and Synthesis in Supervision (CFASS)	English	Clinical psychology master's students	Self-administration	Not reported	Current clinical supervision	1	5	6-point Likert scale	Total score	Available in the methods section
Type of Information Sought	English	Medical residents	Self-administration	Not reported	Not specified	2	10	5-point Likert scale	Total scores of each factors	Available in the results section
Motives of Feedback Seeking - German	German	Medical residents	Self-administration	Not reported	Not specified	4	16	5-point Likert scale	Total scores of each factors	Available as an appendix
Frequency of Feedback Seeking - German	German	Medical residents	Self-administration	Not reported	Not specified	1	6	5-point Likert scale	Total score	Available as an appendix
Dental Students' Perceptions on the Quality of Clinical Feedback	English	Dental students in clinical rotations	Self-administration	Not reported	Not specified	6	13	5-point Likert scale	Individual scores of each items	Available in the results section
Feedback Survey Instrument	English	Medical students in clinical rotations	Self-administration	Not reported	Current clinical rotation	3	19	5-point Likert scale and open ended questions	Individual scores of each items	Available as an appendix
Dental Students' Perceptions towards Feedback during Clinical Training	English	Dental students in clinical rotations	Self-administration	Not reported	Not specified	3	17	4, 5-point Likert scale and open ended questions	Individual scores of each items	Available in the results section
Quality Feedback Inventory	English	Nursing students in clinical rotations	Self-administration	Not reported	Recently finished clinical rotation	3	23	5-point Likert scale	Not described	Available in the results section
Feedback Literacy and Attitudes Survey	English	Year 1 Osteopathy students	Self-administration	Not reported	Not specified	4	21	5-point Likert scale	Total score	Available as an appendix

### 3.4 Constructs of instruments

The included instruments have various constructs, listed in Table 3. Further, these constructs have been categorized into assessments of knowledge, perceptions, and/or behaviours. Ten of the constructs focus on how students perceive the feedback process, two focus on the students' behaviours and only one require students to report their perceptions and behaviours. In addition, nine instruments interpret only teachers as feedback providers. However, the FEEDME instruments require respondents to evaluate any individual that provides feedback, whereas the ‘Feedback Literacy and Attitudes Survey’ asks participants to provide perceptions of their teachers and peers. Twelve out of the thirteen instruments ask respondents to reflect on feedback generally with the exception for ‘Medical Students’ Perceptions on the Quality of Feedback’, wherein different areas of competency (e.g., history taking, physical examination) were specified explicitly. In terms of Carless and Boud's features of feedback literacy [10], all instruments covered feedback appreciation, but only one covered all four features.

**Table 3:** Constructs of instruments.

Instrument	Concept Elicitation	Construct	Category	Feedback Provider	Features of Feedback Literacy			
					AF	MJ	ME	TA
Medical Students' Perceptions on the Quality of Feedback	Literature Review	Perception of frequency and quality of feedback in clinical rotations	Perception	Teacher	✓			
FEEDME Provider	Literature Review and Cognitive Interviews	Perception of any feedback provider	Perception	Any provider	✓		✓	✓
FEEDME Culture	Literature Review and Cognitive Interviews	Perception of feedback culture in a clinical rotation or institution	Perception	Any provider	✓	✓		✓
FEEDME Culture - Spanish	Literature Review and Cognitive Interviews	Perception of feedback culture in a clinical rotation or institution	Perception	Any provider	✓	✓		✓
Corrective Feedback Acceptance and Synthesis in Supervision (CFASS)	Literature Review	Reception of corrective feedback from clinical supervisors	Behaviour	Teacher	✓		✓	✓
Type of Information Sought	Literature Review	Motives for seeking feedback (improvement and or validation)	Perception	Teacher	✓			✓
Motives of Feedback Seeking - German	Literature Review	Motives for seeking feedback (improvement, validation, ego protection and/or impression defence)	Perception	Teacher	✓			✓
Frequency of Feedback Seeking - German	Literature Review	Frequency of seeking feedback through observation or inquiry	Behaviour	Teacher	✓			✓
Dental Students' Perceptions on the Quality of Clinical Feedback	Literature Review	Perception of feedback quality in a clinical rotation	Perception	Teacher	✓	✓		
Feedback Survey Instrument	Not reported	Attitudes and experiences receiving feedback in a clinical rotation	Perception	Teacher	✓	✓		✓
Dental Students' Perceptions towards Feedback during Clinical Training	Literature Review	Perception of the importance, process, and content of feedback during clinical rotations	Perception	Teacher	✓			✓
Quality Feedback Inventory	Scoping Review	Perception on growth with feedback, factors of effective feedback and goals of feedback	Perception	Teacher	✓		✓	
Feedback Literacy and Attitudes Survey	Literature Review	Understanding, capacity, and disposition to process feedback information and apply it for improvement	Perception and behaviour	Teacher and peers	✓	✓	✓	✓

*Abbreviations.* C: Cognitive domain; P: Psychomotor domain; A: Affective domain; AF: Appreciating feedback; MJ: Making judgments; ME: Managing emotions; TA: Taking actions.

### 3.5 Psychometric properties of instruments

In terms of psychometric aspect as presented in Table 4, all instruments have evidence on its development but mostly has inadequate quality, and almost all except three (Medical Students' Perceptions on the Quality of Feedback, Dental Students' Perceptions on the Quality of Clinical Feedback, Feedback Survey Instrument) reported its evidence on internal consistency. Several other properties were also investigated such as structural validity (all FEEDME variations, Type of Information Sought, Quality Feedback Inventory) and convergent validity with hypothesis testing (Corrective Feedback Acceptance and Synthesis in Supervision, Type of Information Sought, Motives of Feedback Seeking, Frequency of Feedback Seeking) but the quality of the latter properties is ‘doubtful’. No evidence of other properties was found.

**Table 4:** COSMIN rating of instruments.

Instrument	Content Validity (Development and Further Studies)			Structural Validity			Internal Consistency			Cross-cultural Validity/Measurement Invariance			Test-Retest Reliability			Measurement Error			Criterion Validity			Hypotheses Testing			Responsiveness		
	M	R	E	M	R	E	M	R	E	M	R	E	M	R	E	M	R	E	M	R	E	M	R	E	M	R	E
Medical Students' Perceptions on the Quality of Feedback	I	±	VL																								
FEEDME Provider	D	+	L	A	+	M	V	+	H																		
FEEDME Culture	D	+	L	A	+	M	V	+	H																		
FEEDME Culture - Spanish	I	±	VL	A	+	M	V	+	H																		
Corrective Feedback Acceptance and Synthesis in Supervision (CFASS)	I	±	VL		+	M	V	+	H													D	?	L			
Type of Information Sought	I	±	VL	A	+	M	V	+	H													D	–	L			
Motives of Feedback Seeking - German	I	±	VL				V	?	H													D	?	L			
Frequency of Feedback Seeking - German	I	±	VL				V	?	H													D	?	L			
Dental Students' Perceptions on the Quality of Clinical Feedback	I	±	VL																								
Feedback Survey Instrument	I	±	VL																								
Dental Students' Perceptions towards Feedback during Clinical Training	I	±	VL				V	?	H																		
Quality Feedback Inventory	I	±	VL	A	+	M	V	+	H																		
Feedback Literacy and Attitudes Survey	I	±	VL				V	?	H																		

*Note.* The measurement properties assessed follows the COSMIN Risk of Bias Checklist [39]. Empty cells represent measurement properties that have not been reported in the studies.

*Abbreviations*. M, Methodology; R, Result; E, Evidence; V, Very good; A, Adequate; D, Doubtful; I, Inadequate; +, Sufficient; ±, Inconsistent; ?, Indeterminate; -, Insufficient; H, High; M, Moderate; L, Low; VL, Very Low.

Based on the COSMIN guidelines, eleven instruments had ‘insufficient’ content validity and the rest were ‘doubtful’. Overall, all instruments (except FEEDME Culture and FEEDME Provider) had ‘inconsistent’ rating with ‘very low’ quality evidence. Nine studies (all except the FEEDME variations) elicited the construct through a literature review alone, without assessing the comprehensiveness and comprehensibility of the instruments. Meanwhile, studies that collected data qualitatively (i.e. cognitive interviewing) [49,50] failed to clearly report the procedures involved. Additionally, none of the instruments had undergone external validation.

Only five of the instruments (all FEEDME variations, Type of Information Sought, Quality Feedback Inventory) provided evidence of their structural validity. The methodological quality was ‘adequate’, with ‘sufficient’ results and a ‘moderate’ level of evidence. These ‘adequate’ results were obtained because the studies performed exploratory factor analysis with sufficient participants.

Ten studies have reported the internal consistency of their instruments in the form of Cronbach's alpha (α > 0.70). The methodological quality was found to be ‘very good’, with ‘sufficient’ results of high quality. However, four instruments (Motives of Feedback Seeking – German, Frequency of Feedback Seeking – German, Dental Students' Perceptions towards Feedback during Clinical Training and Feedback Literacy and Attitudes Survey) had ‘indeterminate’ results as no factor analysis was reported.

Next, hypothesis testing was performed by four of the 13 instruments. The methodological quality was found to be ‘doubtful’, as the constructs in comparison did not have methodological rigour as defined by the COSMIN guidelines. The results were considered ‘indeterminate’ for most instruments, as no hypotheses were formulated prior to testing convergent validity. All these results had ‘low’ evidence due to a ‘doubtful’ methodological quality.

Hence, the Type of Information Sought is the instrument with the most properties investigated. However, the FEEDME instrument is the instrument with the best evidence available especially on its Provider and Culture version. Two instruments, the Frequency of Feedback Seeking, and Quality Feedback Inventory, although has lesser properties explored than the previous two, but more investigated than other instruments.

## 4 Discussion

### 4.1 Utility of instruments

This systematic review had identified and examined psychometric properties of various feedback literacy instruments in the health professions education. In terms of implementability, most instruments are available in English and can be administered readily to students. The majority are self-administered with a manageable number of items. In addition, many instruments are publicly available as attachments. Therefore, the instruments are freely accessible for use by researchers and educators and should be used to further the discussion on feedback literacy. However, upon assessment of the methodological quality using the COSMIN guidelines [39], a need remains for improved instruments as the assessment found none of the instruments had sufficient quality.

### 4.2 Constructs of instruments

Although feedback literacy is a concept first formally conceptualized by Carless and Boud in 2018 [10,22], the practice of involving and empowering learners to share responsibility in the feedback process is not novel. This is evident by the examination of research articles published before the conceptualization, as early as 2007 [20,21,51,52]. Although majority of the instruments did not develop their instruments based on the concept of feedback literacy outlined by Carless and Boud [10] the constructs within these and more recent studies converge on the use of feedback literacy in learning. They can generally be divided into assessment of students' perception of the feedback process [16,17,[49], [50], [51],^53^,^54^] and assessment of students’ behaviours in receiving feedback [17,52,55]. Overall, the included instruments have variable constructs for different contexts.

The four features of feedback literacy [10] were each represented across the studies. However, only one of thirteen (Feedback Literacy and Attitudes Survey) provided a measurement for each feature and ‘appreciating feedback’ was overrepresented compared to the other three features. It is thus apparent that instruments focus on students' appreciation and effectiveness of the feedback process, but are limited in their ability to measure their role in feedback. For example, ‘making judgements’ and ‘taking actions’ are equally important for learners to effectively participate in the feedback process [10]. Interestingly, the least measured feature is ‘managing affect’, which is an integral part of internalizing feedback [10,56]. Ideally, instruments should assess all four features of feedback literacy to comprehensively evaluate students' behaviour and perceptions of receiving feedback. In this way, educators can identify areas of strength and improvement.

### 4.3 Application of instruments in health professions education

In the context of health professions education, all the instruments reviewed were developed for students of health professions education. However, there is a difference in the approach of feedback literacy measurement. Some instruments assessed feedback literacy in a broad manner, using terminology such as “… feedback helped me improve my performance” [49] and “… feedback is important for my learning …” [17]. On the other hand, there are instruments where the items are worded more specifically such as “… importance of feedback on history taking/communication skills/clinical examinations …” [51]. As health professions educations carries unique contexts in its teaching and learning [5,6,8], educators should make an informed-choice between instruments using different approaches that would satisfy their needs better.

Current health profession education strategies encourage competency-based education [[57], [58], [59]], which can be facilitated through feedback literacy. The feedback dialogues should cover all three domains of competencies explicitly to ensure clear communication. However, most feedback literacy instruments evaluated here do not distinguish between knowledge, skill, and attitude. Only one instrument (Medical Students' Perceptions on the Quality of Feedback) differentiated between these domains of competencies. It is important to understand students’ interpretation of feedback provided for each of these domains, as these provide an opportunity for them to target these areas.

It is important to note that the instruments focus on the traditional role of teachers as the sole source of feedback. The exclusion of other stakeholders as feedback providers in health professions education (e.g. peers, other healthcare staffs, patients) in these instruments is a missed opportunity to explore feedback literacy holistically.

### 4.4 Psychometric properties of instruments

Only the FEEDME variations and Type of Information Sought instruments have a considerable number of psychometric properties available; however, it is considered minimum. Having evidence on construct and internal structure is considered a basic requirement for an instrument and investigation on hypothesis testing is a preliminary step [60]. Other properties should be investigated especially the test-retest reliability and responsiveness Thus, subsequent research studies should focus on developing and refining feedback literacy instruments that can be adapted to the many contexts within health professions education. As outlined by the COSMIN guidelines, a robust methodology is required to ensure valid and reliable measurements. Further, instruments should measure each of the four features of feedback literacy [10] to increase utility. These features are important as a guide for instrument development so that measurement of feedback literacy would be comprehensive, and evidence based. Specifically, instruments should be designed to measure the actions and behaviours of students when receiving feedback, in addition to their perceptions and attitudes [10,11].

## 5 Limitations, strengths, and future directions

Like other systematic reviews, this study was subject to publication bias [[61], [62], [63]] and language bias [62,64]. This review was limited to examining the utility, constructs, and psychometric properties of instruments, without discussing the advantages, disadvantages, or the effectiveness of them. Nonetheless, the present review exhaustively searched multiple databases and performed extensive manual searches to ensure a comprehensive compilation of available feedback literacy instruments for health professions students. As discussion surrounding feedback literacy grows, this review serves as a timely guide for health profession researchers in making informed choices before assessing student feedback literacy. The review was conducted and reported according to well-established and widely used guidelines [25,37,39] to ensure a quality review. Inclusion of instruments that did not explicitly measure feedback literacy but share identical features may further enrich the discussion and conceptualization of feedback literacy for health profession students. Measurements from these instruments may assist stakeholders and institutions in better understanding learners’ behaviours and attitudes when it comes to receiving feedback. Nevertheless, there remains a significant gap in the measurement of feedback literacy. The development and validation of feedback literacy instruments should fit the specific contexts within health profession education. Future research should apply a rigorous methodology to produce a valid and reliable student feedback literacy instrument. Other considerations include a broader exploration of contemporary learning and assessment measures for health profession education.

## Data availability statement

No datasets were generated or analysed during the current study.

## CRediT authorship contribution statement

**Mohamad Nabil Mohd Noor:** Writing – review & editing, Writing – original draft, Visualization, Resources, Methodology, Investigation, Formal analysis, Data curation, Conceptualization. **Sahar Fatima:** Writing – review & editing, Visualization, Resources, Methodology, Investigation, Data curation, Conceptualization. **Jessica Grace Cockburn:** Writing – review & editing, Writing – original draft, Visualization, Supervision, Methodology, Formal analysis, Conceptualization. **Muhammad Hibatullah Romli:** Writing – review & editing, Visualization, Resources, Methodology, Investigation, Formal analysis, Conceptualization. **Vinod Pallath:** Writing – review & editing, Visualization, Conceptualization. **Wei-Han Hong:** Writing – review & editing, Visualization, Conceptualization. **Jamuna Vadivelu:** Writing – review & editing, Visualization, Conceptualization. **Chan Chong Foong:** Writing – review & editing, Writing – original draft, Visualization, Supervision, Methodology, Funding acquisition, Formal analysis, Conceptualization.

## Declaration of competing interest

The authors declare the following financial interests/personal relationships which may be considered as potential competing interests:Chan Choong Foong reports financial support was provided by Ministry of Higher Education Malaysia. If there are other authors, they declare that they have no known competing financial interests or personal relationships that could have appeared to influence the work reported in this paper.
