# Supplementary material for: Systematic review of feedback literacy instruments for health professions students
Source: Heliyon. 2024 May 10;10(10):e31070. doi: 10.1016/j.heliyon.2024.e31070 (PMC11133658; doi:10.1016/j.heliyon.2024.e31070)
Supplement: Multimedia component 1 [file mmc1.docx]

The electronic databases included in the review were Web of Science, Scopus, Cumulative Index to Nursing and Allied Health Literature (CINAHL) Complete, Medline, Psychology and Behavioural Sciences Collection by EBSCO Host, and Education Research Complete by EBSCO Host. Selected keywords were chosen based on the PICO format with added synonyms or related terms based on previous research reviews or MeSH (Medical Subject Heading) terms to expand the reach of the electronic search. In addition, a pilot search was conducted to identify keywords that could retrieve research studies discussing feedback literacy or any of its features, regardless of publication year. The search string for each database is as follows:

**Web of Science** - (((TS=(medic* OR health OR "health science*" OR clinic* OR nurs* OR biomedic* OR pharma* OR nutrition OR dietetic* OR dental OR dentist* OR "allied health" OR "occupational health" OR "environmental health" OR "occupational therap*" OR physiotherap* OR "phsyical therap*" OR "speech therap*" OR "speech language phatolog*" OR "occupational safety" OR psycholog* OR audiolog* OR forensic* OR radiotherap*)) AND TS=("feedback literacy" OR "feedback recepti*" OR "feedback receiv*" OR "receiving feedback" OR "feedback orientation" OR "feedback perception" OR "perception of feedback" OR "feedback conception" OR "feedback seeking" OR "feedback behavio*" OR "feedback attitude" OR "feedback culture" OR "feedback dialogue" OR "feedback acceptance" OR "perspectives of feedback" OR "feedback perspectives" OR "feedback practice")) AND TS=(validit* OR reliabilit* OR sensitivit* OR precision OR specificit* OR responsiveness OR psychometri* OR "coefficient of variation" OR "cognitive interview" OR comprehensi* OR "factor analysis" OR "internal consistenc*" OR "reproducibilit*" OR Cronbach OR "structural equation model*" OR "measurement invariance")) AND TS=(instrument* OR measur* OR test OR assessment OR evaluat* OR tool OR questionnaire OR survey) and Preprint Citation Index (Exclude – Database) and Article (Document Types) and English (Languages)

**Scopus** - ( TITLE-ABS-KEY ( medic* OR health OR "health science*" OR clinic* OR nurs* OR biomedic* OR pharma* OR nutrition OR dietetic* OR dental OR dentist* OR "allied health" OR "occupational health" OR "environmental health" OR "occupational therap*" OR physiotherap* OR "phsyical therap*" OR "speech therap*" OR "speech language phatolog*" OR "occupational safety" OR psycholog* OR audiolog* OR forensic* OR radiotherap* ) AND TITLE-ABS-KEY ( "feedback literacy" OR "feedback recepti*" OR "feedback receiv*" OR "receiving feedback" OR "feedback orientation" OR "feedback perception" OR "perception of feedback" OR "feedback conception" OR "feedback seeking" OR "feedback behavio*" OR "feedback attitude" OR "feedback culture" OR "feedback dialogue" OR "feedback acceptance" OR "perspectives of feedback" OR "feedback perspectives" OR "feedback practice" ) AND TITLE-ABS-KEY ( validit* OR reliabilit* OR sensitivit* OR precision OR specificit* OR responsiveness OR psychometri* OR "coefficient of variation" OR "cognitive interview" OR comprehensi* OR "factor analysis" OR "internal consistenc*" OR "reproducibilit*" OR cronbach OR "structural equation model*" OR "measurement invariance" ) AND TITLE-ABS-KEY ( instrument* OR measur* OR test OR assessment OR evaluat* OR tool OR questionnaire OR survey ) ) AND ( LIMIT-TO ( DOCTYPE , "ar" ) ) AND ( LIMIT-TO ( LANGUAGE , "English" ) ) AND ( LIMIT-TO ( SRCTYPE , "j" ) )

**CINAHL Complete** - TX ( medic* OR health OR "health science*" OR clinic* OR nurs* OR biomedic* OR pharma* OR nutrition OR dietetic* OR dental OR dentist* OR "allied health" OR "occupational health" OR "environmental health" OR "occupational therap*" OR physiotherap* OR "phsyical therap*" OR "speech therap*" OR "speech language phatolog*" OR "occupational safety" OR psycholog* OR audiolog* OR forensic* OR radiotherap* ) AND TX ( "feedback literacy" OR "feedback recepti*" OR "feedback receiv*" OR "receiving feedback" OR "feedback orientation" OR "feedback perception" OR "perception of feedback" OR "feedback conception" OR "feedback seeking" OR "feedback behavio*" OR "feedback attitude" OR "feedback culture" OR "feedback dialogue" OR "feedback acceptance" OR "perspectives of feedback" OR "feedback perspectives" OR "feedback practice" ) AND TX ( validit* OR reliabilit* OR sensitivit* OR precision OR specificit* OR responsiveness OR psychometri* OR "coefficient of variation" OR "cognitive interview" OR comprehensi* OR "factor analysis" OR "internal consistenc*" OR "reproducibilit*" OR Cronbach OR "structural equation model*" OR "measurement invariance" ) AND TX ( instrument* OR measur* OR test OR assessment OR evaluat* OR tool OR questionnaire OR survey )

Expanders: Apply related words, Apply equivalent subjects

Limiters: English Language, Peer Reviewed, Research Article, Language: English, Publication Type: Journal Article

**Medline / Psychology and Behavioral Sciences Collection / Education Research Complete** - ( medic* OR health OR "health science*" OR clinic* OR nurs* OR biomedic* OR pharma* OR nutrition OR dietetic* OR dental OR dentist* OR "allied health" OR "occupational health" OR "environmental health" OR "occupational therap*" OR physiotherap* OR "phsyical therap*" OR "speech therap*" OR "speech language phatolog*" OR "occupational safety" OR psycholog* OR audiolog* OR forensic* OR radiotherap* ) AND ( "feedback literacy" OR "feedback recepti*" OR "feedback receiv*" OR "receiving feedback" OR "feedback orientation" OR "feedback perception" OR "perception of feedback" OR "feedback conception" OR "feedback seeking" OR "feedback behavio*" OR "feedback attitude" OR "feedback culture" OR "feedback dialogue" OR "feedback acceptance" OR "perspectives of feedback" OR "feedback perspectives" OR "feedback practice" ) AND ( validit* OR reliabilit* OR sensitivit* OR precision OR specificit* OR responsiveness OR psychometri* OR "coefficient of variation" OR "cognitive interview" OR comprehensi* OR "factor analysis" OR "internal consistenc*" OR "reproducibilit*" OR Cronbach OR "structural equation model*" OR "measurement invariance" ) AND ( instrument* OR measur* OR test OR assessment OR evaluat* OR tool OR questionnaire OR survey )

Expanders: Apply related words, Apply equivalent subjects

Limiters: English Language, Human, Language: English, Scholarly (Peer Reviewed) Journals
